# NaOH-Debittering Induces Changes in Bacterial Ecology during Table Olives Fermentation

**DOI:** 10.1371/journal.pone.0069074

**Published:** 2013-07-31

**Authors:** Luca Cocolin, Valentina Alessandria, Cristian Botta, Roberta Gorra, Francesca De Filippis, Danilo Ercolini, Kalliopi Rantsiou

**Affiliations:** 1 Dipartimento di Scienze Agrarie, Forestali e Alimentari, Università degli Studi di Torino, Grugliasco, Italy; 2 Dipartimento di Agraria, Università degli Studi di Napoli Federico II, Portici, Italy; Teagasc Food Research Centre, Ireland

## Abstract

Limited information is available on the impact of the NaOH treatment on table olive fermentations, and for this reason a polyphasic approach has been adopted here to investigate its effect on the fermentation dynamics and bacterial biodiversity. The microbial counts of the main groups involved in the transformation have not shown any differences, apart from a more prompt start of the fermentation when the olives were subjected to the NaOH treatment. The data produced by culture-independent analyses highlighted that the fermentation of table olives not treated with NaOH is the result of the coexistence of two different ecosystems: the surface of the olives and the brines. A sodium hydroxide treatment not only eliminates this difference, but also affects the bacterial ecology of the olives to a great extent. As proved by high-throughput sequencing, the fermentation of the olives not treated with NaOH was characterized by the presence of halophilic bacteria, which were substituted by *Lactobacillus* at the later stages of the fermentation, while enterobacteria were dominant when the olives were treated with sodium hydroxide. Higher biodiversity was found for *Lactobacillus plantarum* isolated during untreated fermentation. Different biotypes were found on the olive surface and in the brines. When the debittering process was carried out, a decrease in the number of *L. plantarum* biotypes were observed and those originating from the surface of the olive did not differentiate from the ones present in the brines.

## Introduction

Table olives are an important fermented food in Mediterranean countries and they constitute a fundamental food production sector. Olives are the fruit that is produced by the olive tree (*Olea europea*), a species that is successfully cultivated in all Mediterranean countries. Estimations made by the International Olive Council for the 2011/2012 campaign indicate a world production of 3.1 million tons, with a 3% increase compared to the previous year. About 3 quarters of this production is from the European Union (EU), with a production of 2.18 million tons. Spain is the first producing country in the EU, producing 1.35 million tons (62% of the production), and this is followed by Italy (20%), Greece (14%), Portugal (3%) and other EU countries (1%) [1].

The olive fruit, which has a strong bitter taste that comes from glucoside oleuropein, is subjected to a number of transformations, through fermentation, which make them edible, stable and safe. Most table olive fermentation processes start spontaneously and are influenced to a great extent by the olive cultivar itself, its indigenous microbiota [2] and methodological factors, such as fermentation temperature and the salt concentration in the brines [3]. It is widely accepted that the main microbiota responsible for table olive fermentations are lactic acid bacteria (LAB) members, namely *Lactobacillus* spp., and yeasts [4], [5].

There are several ways of treating table olives. However, the green Spanish type and the Greek type are those that account for most of the world's production, with the Spanish type covering about 60% [6]. The first method consists of treating the fruit with a diluted NaOH solution (2–3%) to reduce the bitterness, through the degradation of oleuropein and polyphenols, but also to increase the permeability of the olive pericarp. The debittering treatment is followed by a water wash to remove the excess alkali. The olives are then placed under brine (initial concentration of 8–12%), where they undergo lactic acid fermentation [7]. Instead, in the Greek production, the natural or untreated olives (green or naturally black) are directly brined after picking. In brine, the olives undergo a mixed-acid fermentation until they at least partially lose their bitterness. The fermentation period therefore depends on the physico-chemical conditions, such as the type of cultivar, the salt content and the temperature [3], [4], [6].

The scientific community recognizes that the use of methods that rely on the cultivation of microorganisms (culture-dependent techniques) do not offer a complete profile of the microbial diversity that is present in a specific ecosystem [8]. Culture-independent techniques have attracted the attention of many scientists from different investigation domains, ranging from environmental microbiology to food fermentation. At present, high-throughput sequencing has emerged as a new culture-independent tool to quantitatively investigate the structure of microbial communities, several applications can be already found in food microbiology [9]–[14] and the related critical aspects have been recently reviewed [15]. This and other molecular techniques were employed here for the evaluation of the microbial diversity during olives fermentation.

The aim of this study was to evaluate the changes in bacterial ecology in table olives during fermentation and to assess the effect of NaOH treatment on the bacterial ecology and dynamics.

## Methods

### Table olive fermentations

In November 2009, two table olive fermentations were conducted in duplicate and studied in a small enterprise (SME) in Sicily, Italy. Vats containing 140 kg of the Nocellare etnea variety were prepared. Two of them contained olives treated with NaOH (1%, w/v), while the other two were loaded with olives rinsed with tap water only. In this study, a short 30 min lye treatment was employed, as it is the normal procedure the SME employs in the preparation of the olives prior to fermentation. I should be mentioned that normally this treatment lasts several hours, until the NaOH solution reaches 2/3 of the flesh [16]. Vats were filled with 60 L of brine (8% NaCl, w/v) and the fermentation was carried out at room temperature (without temperature control) for a period of 3 months. The activities described here did not require any specific permission and they were the expression of interest of the SME, which commissioned the study after receiving the funding from the European Commission (FP7/2007–2013), under grant agreement no. 243471- PROBIOLIVES (www.probiolives.eu). The study did not involve endangered or protected species.

### Microbiological analysis and pH measurement

Samples, constituted of 25 g of olives and 50 ml of brines, were collected at 3, 8, 10, 15, 30, 60 and 90 days from the center of the tank, using a sterile dipper. The olives (after a vigorous rinse with Ringer's solution to remove unattached microorganisms and homogenization in 25 ml of Ringer) and brines of both fermentations were sampled separately. Appropriate decimal dilutions were prepared for microbial enumeration of the predominant populations and plated, in triplicate, on different media: lactic acid bacteria (LAB) on de Man Rogosa Sharp agar (MRS, Oxoid, Milan, Italy) using the double layer technique and incubated at 30°C for 48 h; yeasts and molds on malt extract agar (Oxoid) supplemented with a tetracycline solution (1μg/mL, Sigma-Aldrich, Milan, Italy) incubated at 30°C for 48 h; and enterobacteria on Violet Red Bile Glucose Agar (Oxoid) incubated at 37°C for 24 h. After the incubation time, the colonies were counted and the means and standard deviations were calculated. A total of 10 colonies were randomly isolated from the MRS plates at each sampling point and, after purification and growth in MRS broth for 24 h at 30°C, they were stored at −80°C with 20% (w/v) glycerol (Sigma).

The pH of the brine was measured, for both types of fermentation, using a Basic 20 pH meter (Crison, Modena, Italy).

### Identification of the LAB isolates

After DNA extraction, as described by Cocolin et al. [17], and normalization at 100 ng/ µL, the LAB isolates were identified by multiplex PCR analysis of the *rec*A gene with species-specific primers for *Lactobacillus pentosus*, *L. plantarum* and *Lactobacillus paraplantarum*, according to the protocol described by Torriani et al. [18].

### Rep-PCR fingerprinting of the *Lactobacillus plantarum* isolates

Rep-PCR was performed on DNA extracted from *L. plantarum* isolates with the single oligonucleotide primer (GTG)_5_ (5′-GTGGTGGTGGTGGTG-3′) [19] using the conditions described by Dal Bello et al. [20]. Rep-PCR products were electrophoresed in a 2% agarose gel for 4 h at a constant voltage of 120 V in a 1X TBE buffer (10 mmol/L Tris-borate, 1 mmol/L EDTA, pH 8.0) and externally stained using ethidium bromide (0.5 mg/mL, Sigma). A 1 Kb DNA ladder (Sigma) was used as a molecular size marker. Rep-PCR profiles were visualized under ultraviolet light, and this was followed by digital image capturing using a CCD UVI pro Platinum 1.1 (Eppendorf, Milan, Italy). The resulting fingerprints were analyzed using the BioNumerics 4.6 software package (Applied Maths, Sint-Martens-Latem, Belgium). Similarity among the digitized profiles was calculated using the Pearson correlation, and an average linkage (UPGMA) dendrogram was derived from the profiles.

### DGGE analysis

One ml of the olives homogenate and 1 ml of brines were subjected to centrifugation at 13,400×*g* at 4°C and direct nucleic acid extraction was performed from the resulting pellets as described by Cocolin et al. [17]. In order to investigate the dominant bacterial species, the variable V3 region of the 16S rRNA gene was amplified with 338f (5′-ACT CCT ACG GGA GGC AGC AGC AG-3′) and 518r (5′- ATT ACC GCG GCT GCT GG-3′) primers. A GC clamp (5′- CGC CCG CCG CGC GCG GCG GGC GGG GCG GGG GCA CGG GGG G-3′) was attached to the 5′ end of primer 338f for DGGE analysis [21]. The PCR and reverse transcription (RT)-PCR reactions were performed in a final volume of 25 µL, as previously described [22]. The Dcode universal mutation detection system (BioRad, Milan, Italy) was used for DGGE analysis. DGGE was carried out as previously described [23], with a gradient from 30 to 60%. Electrophoresis was conducted at 200 V for 5 h (with an initial 10 mins at 80 V) at 60°C in a 1X TAE buffer. Gels were stained for 20 min in 1X TAE containing 1X SYBR Green I (Sigma) and then analyzed under UV using UVI pro platinum 1.1 Gel Software (Eppendorf). The DGGE profiles were subjected to image analysis using the Bionumerics 4.6 software, as described above. DGGE bands of interest were excised, re-amplified by PCR, sequenced [24] and sequence similarities were searched for in the National Center for Biotechnology Information (NCBI) database using nucleotide Basic Local Alignment Search Tool (BLAST) analysis [25].

### Pyrosequencing

DNA (standardized at 20 ng/μl) and cDNA (prepared by reverse transcription of 100 ng of RNA in RT reactions containing random hexamers [Promega, Milan, Italy]) were amplified with Gray28f (5′-TTTGATCNTGGCCTCAG-3′) and Gray519r (5′-GTNTTACNGCGGCKGCTG-3′) primers [26] and a 520 bp fragment of the 16S rRNA gene was generated. Tag-encoded FLX amplicon pyrosequencing analysis was carried out in the premises of the Research and Testing laboratories (RTL, Lubbock, TX, USA) using a Roche 454 FLX instrument with Titanium reagents and procedures. Raw reads were analyzed and filtered by using QIIME 1.6.0 software [27]. In order to guarantee a higher level of accuracy in terms of Operational Taxonomic Units (OTUs) detection, after the split library script performed by QIIME, the reads were excluded from the analysis if they had an average quality score lower than 25, if they were shorter than 250 bp and if there were ambiguous base calls. Sequences that passed the quality filters were subjected to denoising and chimera checking, as previously described [28], [29]. Filtered sequences were then clustered in operational taxonomic units (OTU) with 96.5% identity using USEARCH [30] and queried against a database of high quality sequences derived from NCBI with a distributed BLASTn algorithm [31] as previously described [9], [26]. The abundance (%) of each OTU was calculated on the basis of the number of sequence reads obtained in each sample. The OTU taxonomy table was used to produce a heat map by using the clustering software TMeV v 4.8 [32]. Alpha diversity was evaluated through QIIME to generate rarefaction curves, Good's coverage, Chao1 richness [33] and Shannon diversity indices [34]. The OTU taxonomy table and the sequence phylogenetic tree were used to generate the weighted UniFrac distance matrix [35]. Samples were clustered using UPGMA (Unweighted Pair Group Method with Arithmetic mean, also known as average linkage). Sequences are available at the Sequence Read Archive (SRP019475).

### Statistical analysis

Statistical analysis of the microbial counts during fermentation was performed using the Statistica software package (version 7.1, StatSoft Inc., Tulsa, OK). The Tukey test was used in order to establish any statistical differences by one-way analysis of variance (ANOVA) between NaOH treated and not treated table olive fermentations.

## Results

### Microbial trends determined by plate counts and pH measurement

The counts performed during the fermentations followed in this study are presented in Table 1. The main populations involved in the transformation process were once again confirmed to be LAB and yeasts, which dominated the ecosystem from the very beginning. Significant differences were observed between the NaOH treated and untreated table olive fermentations, especially at the beginning of the transformations (for LAB and yeasts *P*<0.01 and for enterobacteria *P*<0.05 on the olive surface and for enterobacteria *P*<0.01 and yeasts *P*<0.05 in brines, Table 1). The populations present on the surface of the untreated olives were delayed at the beginning of the fermentation and only at day 30 did the LAB and yeasts exceed 10^6^ colony forming units (cfu)/g. In the NaOH treated olives, these counts were already reached at day 15. Such a difference was not so evident in the brines, and in both cases, after 10 days of fermentation, the LAB and yeasts counts were above 10^5^ cfu/mL. The molds only showed an increase in the counts on the olives between day 60 and 90, while they reached final values of about 10^5^–10^6^ cfu/mL in the brines, with the NaOH treated fermentation already showing an increase in molds after day 10, compared to day 30, for the untreated process. No significant differences in mold counts were observed throughout the fermentation period. Like the molds, the enterobacteria also presented a similar trend in all the fermentations. They were not counted from day 60 on the olives, while the transformation without NaOH allowed the elimination of enterobacteria after 60 days in the brines, compared to the 90 days required in the treated fermentations. Significant differences in enterobacteria counts (*P*<0.05) were detected in brines from day 10 to day 30.

**Table 1:** Microbial counts on the olive surfaces and in brines during the fermentation processes examined in this study.

	Days of fermentation						
	3	8	10	15	30	60	90
**Olive surfaces**							
Lactic acid bacteria							
Not treated	2.54 ± 0.08	4.79 ± 1.96	5.51 ± 0.50	4.21 ± 0.73	6.07 ± 0.42	7.06 ± 0.54	5.65 ± 1.44
Treated	6.01 ± 0.04	6.13 ± 0.40	5.69 ± 0.87	7.10 ± 0.44	7.24 ± 0.12	7.22 ± 0.24	6.08 ± 0.13
Sig.^a^	**	NS	NS	NS	*	NS	NS
Enterobacteria							
Not treated	2.91 ± 2.03	5.29 ± 1.53	5.38 ± 0.47	3.92 ± 1.06	5.98 ± 1.53	<5^b^	<5
Treated	5.39 ± 0.10	5.54 ± 1.33	6.20 ± 0.23	7.15 ± 0.80	4.71 ± 1.27	<5	<5
Sig.	*	NS	NS	NS	NS	NS	NS
Yeasts							
Not treated	4.01 ± 0.57	4.33 ± 0.04	4.34 ± 0.23	3.72 ± 0.60	7.25 ± 1.25	7.07 ± 0.69	6.29 ± 1.70
Treated	5.65 ± 0.32	6.27 ± 0.01	4.13 ± 0.80	6.40 ± 0.74	7.66 ± 0.09	7.44 ± 0.33	5.47 ± 0.60
Sig.	**	**	NS	NS	NS	NS	NS
Molds							
Not treated	<50	<50	<50	<50	<50	<50	5.16 ± 1.55
Treated	<50	<50	<50	<50	<50	<50	4.70 ± 0.00
Sig.	NS	NS	NS	NS	NS	NS	NS
**Brines**							
Lactic acid bacteria							
Not treated	4.23 ± 0.11	5.74 ± 0.30	5.81 ± 0.24	6.06 ± 0.25	5.92 ± 0.18	5.44 ± 0.66	6.03 ± 0.35
Treated	5.14 ± 0.92	5.54 ± 0.24	5.67 ± 0.53	5.72 ± 0.37	6.48 ± 0.12	6.57 ± 0.24	6.93 ± 0.49
Sig.	NS	NS	NS	NS	*	*	NS
Enterobacteria							
Not treated	3.00 ± 0.40	3.85 ± 1.29	4.73 ± 0.88	5.27 ± 0.56	4.39 ± 0.34	<1	<1
Treated	5.33 ± 0.49	5.71 ± 0.21	6.36 ± 0.16	6.67 ± 0.04	5.83 ± 0.10	2.04 ± 0.06	<1
Sig.	**	NS	**	**	**	NS	NS
Yeasts							
Not treated	4.22 ± 0.07	5.67 ± 1.04	5.64 ± 0.21	5.89 ± 0.02	6.17 ± 0.29	6.19 ± 0.59	5.37 ± 0.39
Treated	5.09 ± 0.33	6.12 ± 0.18	4.33 ± 0.04	5.88 ± 0.59	6.59 ± 0.19	6.91 ±0.44	6.42 ± 0.26
Sig.	*	NS	NS	NS	NS	NS	*
Molds							
Not treated	<10	<10	<10	<10	4.25 ± 0.49	5.25 ± 0.92	5.35 ± 0.57
Treated	<10	<10	<10	3.62 ± 0.54	5.30 ± 0.43	5.30 ± 1.41	5.14 ± 0.22
Sig.	NS	NS	NS	NS	NS	NS	NS

Values are expressed as average Log_10_ colony forming units per g or ml ± standard deviation.

a , *, ** and NS indicate significance at *P*<0.05, *P*<0.01 and no significant differences, respectively.

b , values presented as “<” are expressed in colony forming units per g or ml.

Both fermentations started with a pH of the brines of 5.7–5.8 and, after a slow decrease in the first 10 days, a drop to values of around 4 – 4.5 was observed. A difference of about 0.5 units of pH was recorded at the end of the fermentation between the two processes. The fermentation of olives treated with NaOH reached pH values of about 4, while the other never went below 4.5 (data not shown).

### Molecular identification and characterization of the LAB isolates

**Figure 1:**
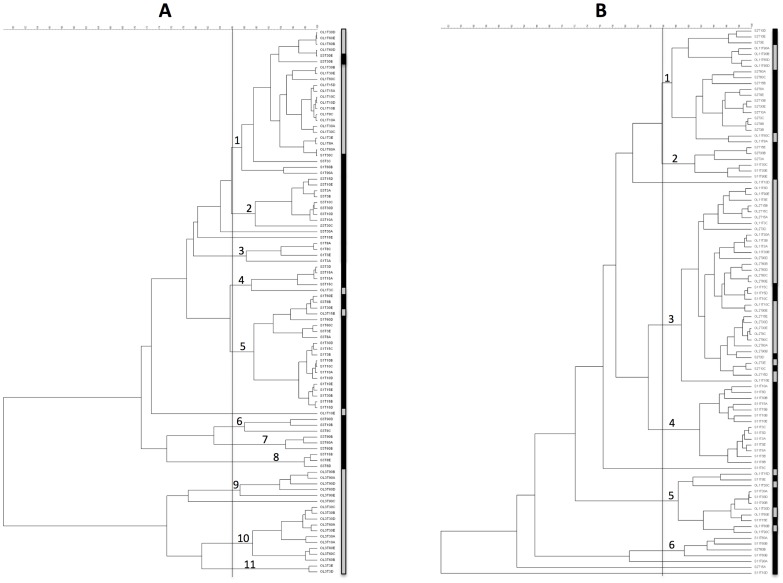
Dendrograms obtained comparing the Rep-PCR profiles of *Lactobacillus plantarum* isolated from the untreated (A) and NaOH treated (B) table olive fermentations. An arbitrary coefficient of similarity of 85% was selected to produce the clusters indicated with numbers. The lines on the right of the dendrograms indicate the origin of the isolates: gray, from the olives; black, from the brines.

A total of 205 LAB were isolated and successfully identified by applying the method suggested by Torriani et al. [18]. The results of the identification are presented in Table 2. The vast majority was identified as *L. plantarum* (187 isolates), while the rest was *L. pentosus* (18 isolates). *Lactobacillus plantarum* was more frequently isolated (94.00%) compared to untreated fermentations (88.57%). The results of the Rep-PCR of *L. plantarum* are presented in Figure 1. The isolates from the surface of the olive and the brines are indicated in the dendrograms with a gray and black box, respectively. Using an arbitrary coefficient of similarity of 85%, isolates from the untreated table olive fermentations (Fig. 1A) clustered in 11 different groups, while in the case of the NaOH treatment, only 6 clusters could be observed (Fig. 1B). The isolates from the untreated fermentations clustered above all according to the source of isolation (as described by the gray and black boxes in Fig. 1). As shown in Figure 1A, *L* *. plantarum* isolated from the surface of the olives formed 4 clusters, namely 1, 9, 10 and 11, among which only in cluster 1 were six isolates from brines included. A homogeneous distribution of the isolates from the olive surfaces and brines was observed in the NaOH treated table olive fermentations. Only clusters 2 and 6, grouping 6 and 4 isolates, respectively, were composed exclusively of *L. plantarum* from the brines (Fig. 1B). Considering the distribution of the *L. plantarum* isolated from the surface of the olives, it can be observed that, in the case of the untreated table olive fermentations, 4 biotypes were detected, compared to the 3 observed in the fermentation with the NaOH treatment.

**Table 2:** Molecular identification of the LAB isolates obtained from treated and untreated table olive fermentations.

		Type of fermentation												Totals	%
		without NaOH treatment						with NaOH treatment							
		olives	%	brines	%	Totals	%	olives	%	brines	%	Totals	%		
*L. pentosus*		2	4.76	10	15.87	12	11.43	1	2.33	5	8.77	6	6.00	**18**	**8.78**
*L. plantarum*		40	95.24	53	84.13	93	88.57	42	97.67	52	91.23	94	94.00	**187**	**91.22**
**Totals**		**42**	**100.00**	**63**	**100.00**	**105**	**100.00**	**43**	**100.00**	**57**	**100.00**	**100**	**100.00**	**205**	**100.00**

Number of isolates and percentages obtained from the surface of the olives and the brines for each treatment are reported in the table.

### DGGE analysis

**Figure 2:**
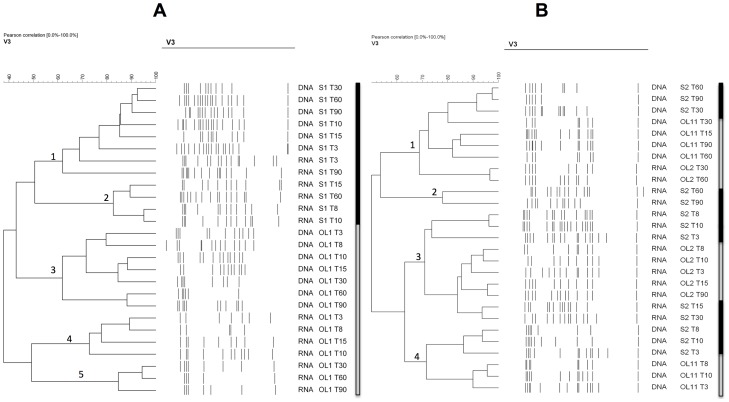
Digitalized DGGE profiles of the DNA and RNA extracted directly from olive surfaces and brines during the fermentation processes. Panel A, untreated table olive fermentation; panel B, NaOH treated table olive fermentation. The identified clusters are indicated with numbers. The lines on the right of the dendrograms indicate the origin of the samples: gray, from the olives; black, from the brines. OL stands for olive surface and S for brine. The day of fermentation and the nucleic acid analyzed is also indicated in the code.

The digitalized profiles of the PCR-DGGE and RT-PCR-DGGE gels, as well as the dendrograms obtained from the cluster analysis performed with the Bionumerics software, are shown in Figure 2. Samples from olive surfaces and brines are indicated with gray and black boxes, respectively. The figure reports one fermentation per type (not treated with NaOH, panel A, and treated with NaOH, panel B). No differences in the profiles were observed for the duplicate fermentations or within each sampling point (data not shown). Generally, the DGGE profiles were characterized by high complexity with some samples containing up to 15 bands. With regards to the fermentations in which the olives were not treated with NaOH, the profiles clustered in 5 groups. Clusters 1 and 2 were related to samples extracted from brines, while clusters 3, 4 and 5 were formed by DGGE profiles produced by nucleic acids on the surface of the olive. Within the clusters of the brines and olives, it was also possible to differentiate samples from DNA and RNA, respectively, which grouped separately. As can be observed, considering the olive surface, clusters 4 and 5 were formed by RNA samples, while cluster 3 grouped the DNA samples. This evidence was not confirmed in the case of the NaOH treated table olive fermentation, in which the dendrogram showed 4 clusters grouping samples regardless of the source and the analyzed nucleic acid. None of the clusters contained samples from only the olive surface or brines, or samples from only the DNA and RNA. Moreover, samples from the beginning and the end of the fermentations generally clustered differently, underlining a change in the bacterial population.

The results of the analysis of the excised and sequenced bands are reported in Table 3. *Marinilactobacillus* and *Lactobacillus* genera were found in both fermentations at DNA and RNA levels, and they represented the most common populations. *Halomonas* and *Salinicola* were mainly detected in fermentations in which the olives were not treated with sodium hydroxide, whereas *Enterobacter, Citrobacter, Acinetobacter* and *Pseudomonas* were mostly observed in the NaOH treated table olive fermentations. The species most frequently detected in DGGE gels were *Marinolactobacillus piezotolerans, L. plantarum, Acinetobacter johnsonii, Citrobacter freundii* and *Pseudomonas mendocina* (data not shown).

**Table 3:** Identification, at a genus level, of selected bands from DGGE gels used to analyze the PCR and RT-PCR products obtained from the amplification of the olive and brine samples.

						Untreated table olive fermentation		NaOH treated table olive fermentation	
						DNA			
Closest described relative	Identity %	Phylum	Class	Order	Family	Olive	Brine	Olive	Brine
*Marinilactibacillus*	99–100	*Firmicutes*	*Bacilli*	*Lactobacillales*	*Carnobacteriaceae*		*		*
*Halomona*s	98–100	*Proteobacteria*	*Gamma*	*Oceanospirillales*	*Halomonadaceae*	*	*		
*Salinicola*	99–100	*Proteobacteria*	*Gamma*	*Oceanospirillales*	*Halomonadaceae*		*		
*Lactobacillus*	99–100	*Firmicutes*	*Bacilli*	*Lactobacillales*	*Lactobacillaceae*	*		*	
*Acinetobacter*	99–100	*Proteobacteria*	*Gamma*	*Pseudomonadales*	*Moraxellaceae*				*
*Enterobacter*	99–100	*Proteobacteria*	*Gamma*	*Enterobacteriales*	*Enterobacteriaceae*			*	*
*Citrobacter*	99–100	*Proteobacteria*	*Gamma*	*Enterobacteriales*	*Enterobacteriaceae*			*	

						RNA			
*Marinilactibacillus*	99–100	*Firmicutes*	*Bacilli*	*Lactobacillales*	*Carnobacteriaceae*		*		*
*Halomonas*	98–100	*Proteobacteria*	*Gamma*	*Oceanospirillales*	*Halomonadaceae*	*	*		*
*Salinicola*	99–100	*Proteobacteria*	*Gamma*	*Oceanospirillales*	*Halomonadaceae*	*	*		
*Lactobacillus*	99–100	*Firmicutes*	*Bacilli*	*Lactobacillales*	*Lactobacillaceae*	*		*	*
*Pediococcus*	100	*Firmicutes*	*Bacilli*	*Lactobacillales*	*Lactobacillaceae*	*			*
*Acinetobacter*	99–100	*Proteobacteria*	*Gamma*	*Pseudomonadales*	*Moraxellaceae*				*
*Enterobacter*	100	*Proteobacteria*	*Gamma*	*Enterobacteriales*	*Enterobacteriaceae*	*	*	*	*
*Pseudomonas*	99	*Proteobacteria*	*Gamma*	*Pseudomonadales*	*Pseudomonadaceae*				*

The presence in the specified sample is indicated by an asterisk.

### Pyrosequencing

The DNA and RNA extracted from the olive and brine samples of both fermentations examined in this study were selected from the DGGE profiles presented in Figure 2 and were subjected to pyrosequencing. The nucleic acids extracted from the samples at the beginning (day 8) and at the end (day 90) of the fermentations were selected because of their different clustering profiles. Moreover, the DNA and RNA from olives treated or not treated with NaOH were also subjected to pyrosequencing at day 3. A total of approximately 74,000 reads were analyzed with an average number of about 3,700 reads per sample. The rarefaction analysis and the diversity indexes indicated that there was a satisfactory coverage of the diversity for all the samples analyzed with Good's coverage values above 97% (Table 4); this result was also confirmed by the analysis of rarefaction curves (Figure S1). Overall, despite the diversity of sequencing depth between samples, the rarefaction analysis indicated that a number of reads above 1,500 per sample was satisfactory to obtain a good coverage (Table 4).

**Table 4:** Number of sequences analyzed, observed diversity and estimated sample coverage for 16S rRNA amplicons from olives fermentations.

		Reads	Observed OTUs	Shannon	Chao1	ESC (%)
**Not treated olives fermentation**						
Olive surfaces						
Day of fermentation	Target					
3	RNA	1697	116	4.68	128.5	98.53
3	DNA	4258	74	2.43	78.33	99.67
8	RNA	5957	70	2.09	80.91	99.73
8	DNA	5199	78	2.92	101.1	99.58
90	RNA	1831	49	2.7	73	99.13
90	DNA	6325	73	2.79	88.55	99.70
Brines						
Day of fermentation	Target					
8	RNA	1894	39	2.09	54.6	98.81
8	DNA	3980	56	2.47	77.38	99.52
90	RNA	2894	68	3.03	83	99.27
90	DNA	3763	77	3.02	89.05	99.39
**NaOH treated olives fermentation**						
Olive surfaces						
Day of fermentation	Target					
3	RNA	1131	40	2.22	57.14	98.59
3	DNA	3306	81	2.6	110.08	99.15
8	RNA	2813	51	2	82.63	99.18
8	DNA	3171	53	2.06	70	99.43
90	RNA	2945	91	3.07	128.71	98.88
90	DNA	1465	80	3.59	107.19	97.95
Brines						
Day of fermentation	Target					
8	RNA	7834	127	3.38	156.75	99.55
8	DNA	2772	101	3.56	125	98.81
90	RNA	5851	82	2.75	111.25	99.54
90	DNA	6428	112	3.27	137.83	99.52

The results obtained for the DNA correlated well with those from RNA. The composition of the bacterial consortium in most of the analyzed samples was the same for both nucleic acids, with a difference of less than 15%. Only in the case of the fermentation in which the olives were not treated with NaOH, was a population of *Pseudomonas* found on the olive surface, but only at an RNA level, with a prevalence of about 50% (Table S1).

**Figure 3:**
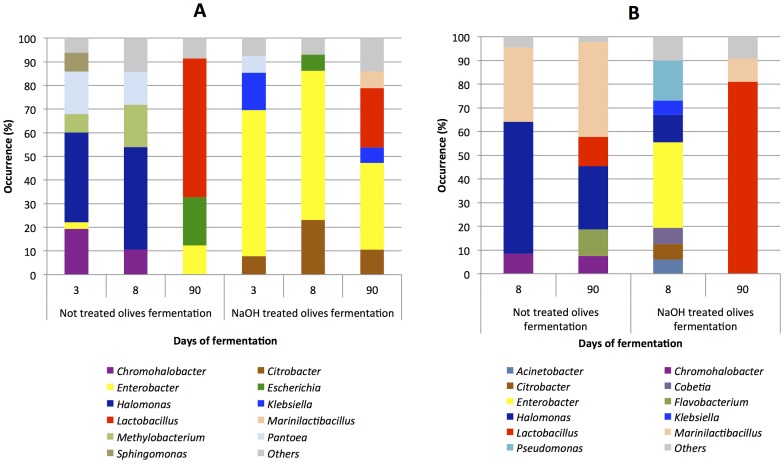
Occurrence (%) of genera obtained by pyrosequencing. Only genera above 5% occurrence are reported. Reference can be made to Table 1S for the entire data set. Panel A, bacterial ecology on the olive surfaces; panel B, bacterial ecology in the brines.

**Figure 4:**
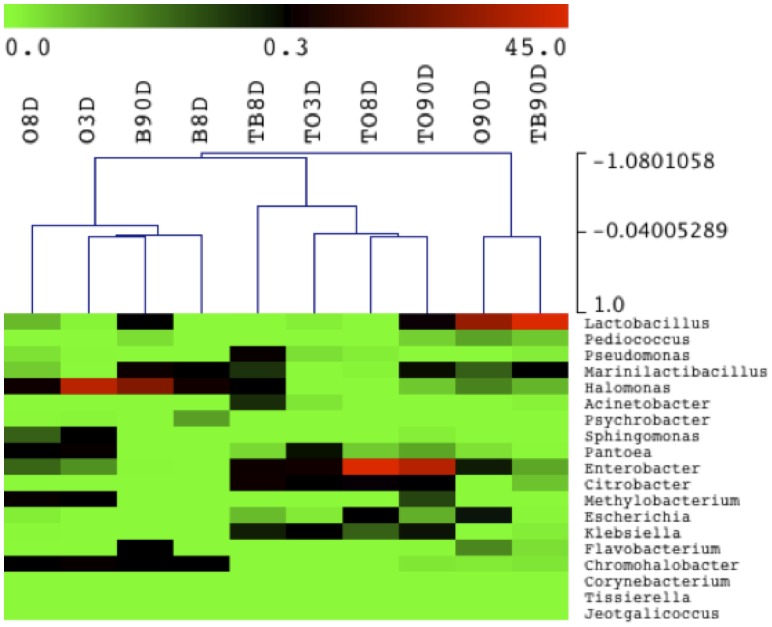
Heat map depicting bacterial diversity and relative abundance in treated (T) and untreated olives (O) and brines (B) during fermentation. Numbers in the samples identity indicate the days of fermentation. Hierarchical dendrogram shows distribution of samples based on average linkage clustering calculated with Pearson correlation. Legend and color scale shown in the upper part of the figure represent colors in the heat map associated with the relative percentage of each OTU within the samples.

The results obtained after sequence identification of the DNA samples are shown in Figure 3, where only OTUs (genus level) that represented at least 5% of the total sequence reads in each sample are shown. Panel A reports the population profiles on the olive surface, while panel B shows the changes in the bacterial ecology of the brines. The entire set of identifications is reported in Table S2. The surface of the olives not treated with NaOH showed a different bacterial colonization in the first 8 days of fermentation compared to the last day of sampling. The initial fermentation stage was characterized by a high level of halophilic bacteria, namely *Chromohalobacter* and *Halomonas*, which represented about 60 and 50% of the total bacterial population at days 3 and 8, respectively. After 90 days of fermentation, the structure of the microbiota changed dramatically, and *Lactobacillus* represented the main bacterial population present on the olive surface (Fig. 3A). This switch in bacterial ecology was not observed on the surface of the olives treated with sodium hydroxide. In fact, enterobacteria already represented the main components of the bacterial consortium at day 3, and basically remained stable until the end of the fermentation. It is worth noticing that the *Lactobacillus* genus was once again only detected at the last sampling point, although with low prevalence (25%) compared to the olives not treated with NaOH (Fig. 3A). Considering the results obtained from the brines (Fig. 3B), a different bacterial ecology could once again be described as being influenced by the NaOH treatment. When the untreated olives were fermented, the brines were characterized by an abundant presence of halophiles throughout the process. *Chromohalobacter, Halomonas* and *Marinilactibacillus* accounted for more than 90% of the total population, and only at day 90 were the *Flavobacterium* and *Lactobacillus* genera detected at percentages of about 10%. A complex bacterial ecosystem was described in the brines, when the olives subjected to the NaOH treatment were fermented. Samples taken at day 8 contained 7 genera, among which *Enterobacter* and *Pseudomonas* were the most numerous. At the end of the fermentation (day 90), an overturned bacterial ecology was observed. This was characterized by a relevant presence (80%) of the *Lactobacillus* genus, which surpassed all the other components of the consortium detected at day 8. *Chromohalobacter salarius and M. piezotolerans* were identified among the halophilic genera, while *L. plantarum* and *L. pentosus* were the main species belonging to the *Lactobacillus* genus, with the first one being the most abundant in all the fermentation processes, confirming the results obtained by traditional isolation and molecular identification. Selecting the minimum incidence of the 0.1% in at least 1 sample we considered 19 OTUs and used their abundance in each sample to generate the hierarchical clustering reported in Figure 4. NaOH treated and untreated manufactures could be clearly distinguished. The untreated olives and brines were characterized by the occurrence of halophilic bacteria regardless of the fermentation time, whereas the NaOH treated samples were separated from the others on the basis of the abundance of *Enterobacteriaceae* such as *Enterobacter*, *Citrobacter, Escherichia* and *Klebsiella*. Finally, untreated olives and treated brines after 90 days of fermentation clustered separately because of the high incidence (above 50%) of *Lactobacillus* (Fig. 4).

## Discussion

The available literature on table olive fermentations is rather extensive and there is international scientific consensus on the fact that LAB and yeasts are the main microbial populations, which are active throughout the transformation process. The significance and the role of these two groups have recently been reviewed in two comprehensive papers by Hurtado et al. [5] for LAB and by Arroyo-Lopez et al. [36] for yeasts.

As pointed out by Botta and Cocolin [37], in the field of table olive fermentations, scientists have only taken advantage of molecular approaches in the last few years and most studies currently available are above all related to the use of molecular methods for the identification of isolated strains [38]–[44]. To the authors' knowledge, culture independent methods have rarely been applied to study the dynamic changes that take place during table olive fermentation. In 2006, Ercolini et al. [45] employed fluorescence in situ hybridization to detect the *L. plantarum* group on olives used in natural fermentations, while Abriouel et al. [46] and Randazzo et al. [47] exploited DGGE to study the diversity of microbial populations in brines during fermentation of Aloreña olives and to assess the influence of inoculated starter cultures on bacterial dynamics during table olive fermentations.

For the first time in this study, olives and brines were treated as different samples in order to investigate the differences in microbial ecology on the fruit and in the liquid brine during fermentation. Appropriate succession of the microbiota and fermentation is reported as necessary to obtain safe and good quality table olives [7]. However, most of the studies focus on the microbiota of the brine, useful for pH drop and food protection, while the microbiota developing on the surface of the olives can be still responsible for changes in the olive texture and sensory properties [48]. So far, the microbiological analysis of table olive fermentations has only been performed by spreading the brines over culture media, and only in a few cases has the microbiological investigation specifically been performed on the olives in order to study the formation of biofilms on the surface of the fruit [2], [16] and to assess adherence of the probiotic strain *L. paracasei* IPMC2.1 inoculated as a starter culture [49]. Therefore in this study olives and brines were handled as two different bacterial ecosystems.

The results obtained from the plate counts have once again highlighted the technological importance of the LAB and yeast populations. When comparing the counts of the fermentations of olives treated or not treated with NaOH, it was interesting to observe a more prompt start of the growth after the sodium hydroxide wash. This evidence was particularly obvious in the case of the olive surface. As can be seen in Table 1, without the NaOH treatment, the counts only reached values of just over 10^6^ cfu/g after 30 days, while those counts were already surpassed at day 8 for the treated olives. More than 90% of the isolated colonies from MRS were identified as *L. plantarum*, regardless of the NaOH treatment. These results correlate well with previous studies of LAB ecology in fermented table olives, in which *L. plantarum* and *L. pentosus* were among the main lactobacilli responsible for the fermentation process [5].

Only a few studies focused on the effect of the NaOH treatment on the olive microbial populations. Arroyo-Lopez et al. [50] reported a total destruction of the initial micobiota in Spanish style processed olives when heavy NaOH treatments were applied, whereas Bevilacqua et al. [51] emphasized that yeasts can survive NaOH processing and colonize olives throughout the fermentation. As far as the brines and the olives are concerned, Hernández et al. [52] found a greater presence of yeast cells in the brine after treatment than on the olive surfaces and also a different predominant species.

The culture independent methods used in this study, i.e. DGGE and pyrosequencing, underlined important differences in the bacterial ecology in NaOH treated or not table olives fermentations. It should be pointed out that while DGGE is only able to pick up major populations, having a limit of detection of about 10^3^ cfu/g or ml [37], pyrosequencing can potentially detect large minor populations and define the relative abundance of the OTUs [15]. Independently from this difference, both methods were able to highlight the changes in the succession of the bacterial populations when the olives are treated with NaOH.

Overall, NaOH modified the composition of the table olive ecosystem to a great extent and promoted a fermentation process that was different, in terms of bacterial species and strain. This evidence could allow us to speculate that the debittering process could influence the number of species present on the surface of olives and affect the biodiversity of the fermentation system. This hypothesis is supported by both the results of the DGGE performed at DNA and RNA levels, in which samples from the olive surfaces and brines clustered in two different groups in untreated table olive fermentations, while they were mixed in the case of the NaOH wash, but also by considering the diversity of *L. plantarum* at a strain level. As observed for the DGGE analysis, in the case of the Rep-PCR characterization, isolates from the untreated olive surfaces again always clustered separately from those in the brines, underlining the presence of different biotypes that colonize the two considered ecosystems (Fig. 1A). This was not found for the *L. plantarum* isolates from the treated table olive fermentation. As reported in Figure 1B, no specific separation was obtained on the basis of the isolation source. Finally, the molecular characterization of the isolates also indicates a reduction in *L. plantarum* biodiversity. This aspect can be seen by simply analyzing the obtained number of clusters, that is, 11 for the untreated and 6 for the treated table olive fermentations, respectively.

The results obtained from pyrosequencing confirmed the different structure of the microbiota of olives and brines during fermentation and definitively strengthened the need to treat them as separate bacterial ecosystems. This was further supported by the UniFrac analysis and clustering of the treated and untreated samples (Figure S2). The effect of the NaOH treatment on the bacterial ecology of the olive surface was remarkable. The halophilic populations found in the untreated olives, were replaced by enterobacteria, which remained stable until the end of the fermentation in the treated olives, and this evidence was also confirmed in the brines at the beginning of the fermentation (Fig. 3 and 4). This is of particular relevance for table olive fermentation because it is well established that enterobacteria are involved in the olive spoilage process described as gas pockets [48], [53]. The presence of halophiles, such as *Marinilactibacillus*, *Halomonas* and *Chromohalobacter,* could be explained by the use of marine salt in the preparation of the brines. Previously, *Halomonas* was detected by means of high-throughput sequencing in the rind of artisanal cheeses produced in Ireland and subjected to a salting process [12], and *Marinilactobacillus* has been used to control *Listeria* spp in the cheese rind ecosystem [54]. Pyrosequencing highlighted relevant differences in the localization of *Lactobacillus*, the main genus of technological importance. *Lactobacillus* was revealed by sequencing only after 90 days of fermentation (Fig. 3). However, *Lactobacillus* colonized the surface of the olives more in untreated than treated fermentations, while it was the dominant population in the brines only when the olives had been treated by NaOH.

This paper has highlighted the effect of NaOH treatment on the bacterial ecology of olives fermentation and the necessity of investigating table olive fermentations as two different ecosystems: the surface of the olives and the brine. Future studies on these transformation processes should take into consideration the evidence reported here, and also try to match the evolution of the microbiota with the changes in sensory quality of the table olives in order to appropriately define the most suitable fermentation conditions to enhance the product quality while assuring its safety.

## Supporting Information

Figure S1 **Rarefaction curves obtained by QIIME for untreated (A) and treated (B) olives and brine samples.** OL stands for olive surface and S for brine. The day of fermentation and the nucleic acid analyzed is also indicated in the code.
(TIFF)

Figure S2 **UPGMA clusters based on weighted UniFrac distance matrix obtained by QIIME for the untreated (A) and treated (B) olives and brine samples.** OL stands for olive surface and S for brine. The day of fermentation and the nucleic acid analyzed is also indicated in the code.
(TIFF)

Table S1 **Differences in the occurrence of the genera obtained by pyrosequencing between DNA and RNA samples.** Positive values mean a higher prevalence at DNA level, negative values indicate a higher prevalence at RNA level.
(DOCX)

Table S2 **Complete results of the identification, at genus level, of the sequences obtained by pyrosequencing and expressed as percentage.**
(DOCX)
