# Supplementary figures and images for: NaOH-Debittering Induces Changes in Bacterial Ecology during Table Olives Fermentation
Source: PLoS One. 2013 Jul 31;8(7):e69074. doi: 10.1371/journal.pone.0069074 (PMC3729808; doi:10.1371/journal.pone.0069074)

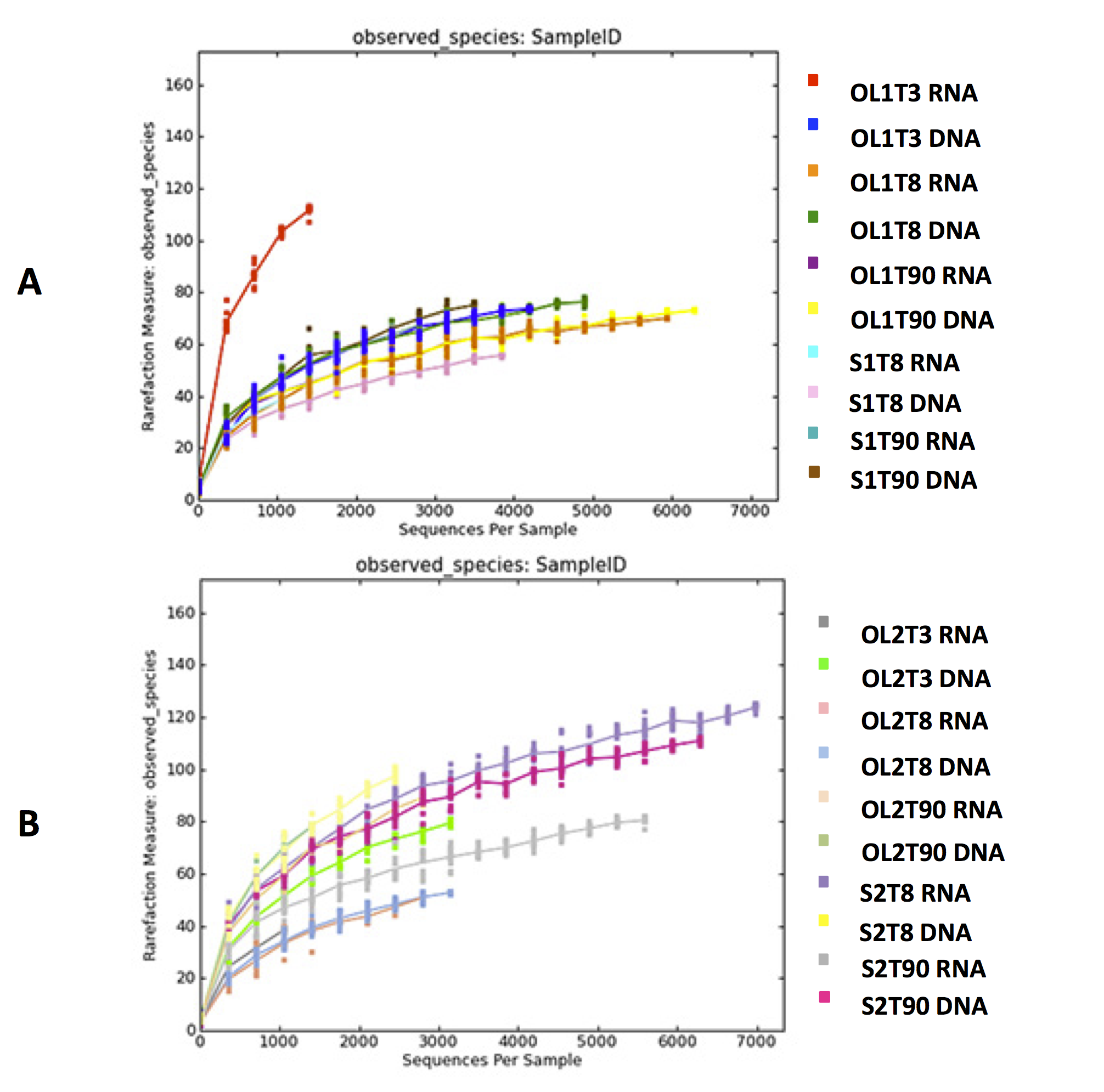

Supplement: Figure S1 — Rarefaction curves obtained by QIIME for untreated (A) and treated (B) olives and brine samples. OL stands for olive surface and S for brine. The day of fermentation and the nucleic acid analyzed is also indicated in the code. (TIFF) [file pone.0069074.s001.tiff]

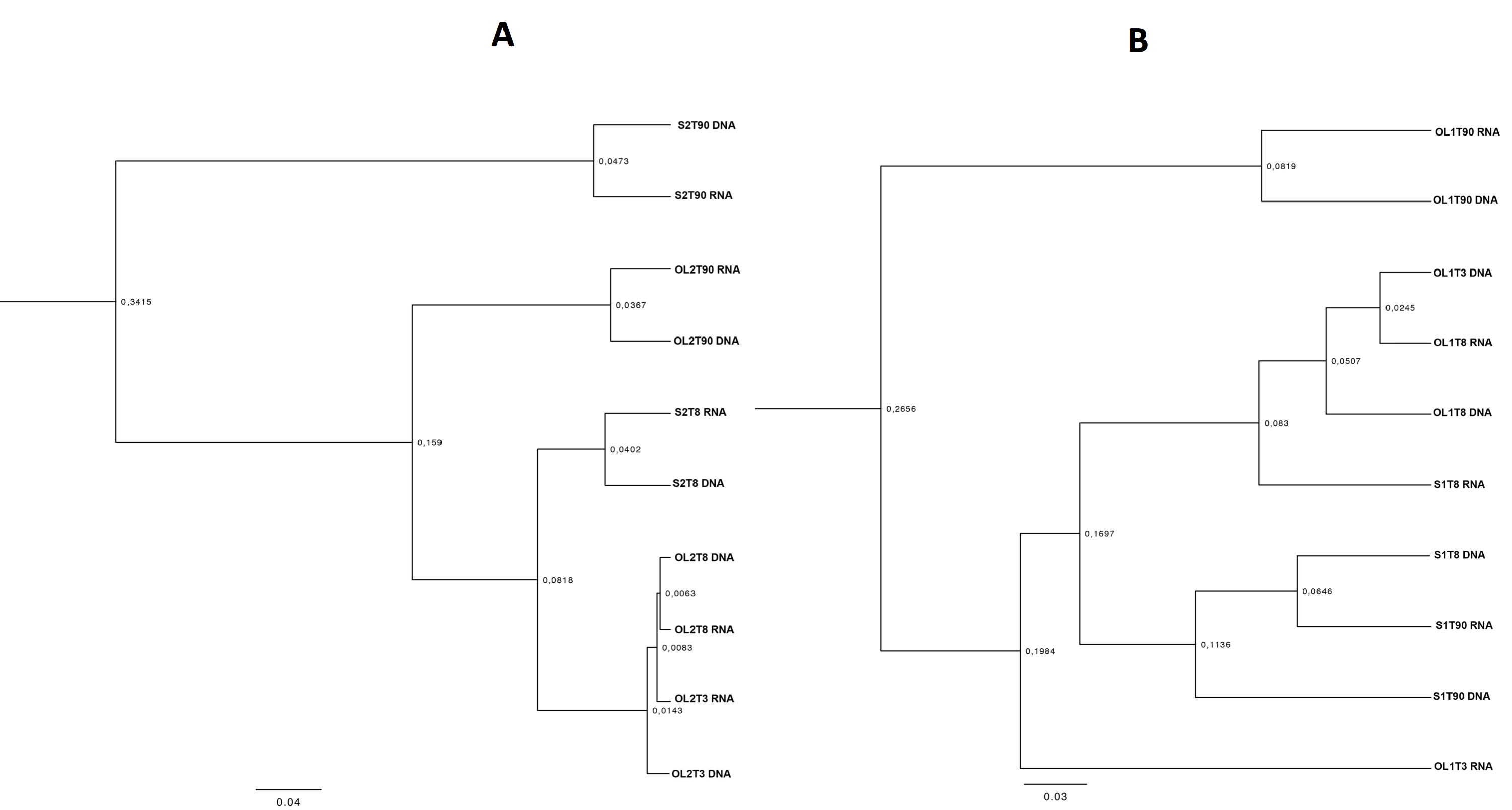

Supplement: Figure S2 — UPGMA clusters based on weighted UniFrac distance matrix obtained by QIIME for the untreated (A) and treated (B) olives and brine samples. OL stands for olive surface and S for brine. The day of fermentation and the nucleic acid analyzed is also indicated in the code. (TIFF) [file pone.0069074.s002.tiff]
