# Supplementary material for: NaOH-Debittering Induces Changes in Bacterial Ecology during Table Olives Fermentation
Source: PLoS One. 2013 Jul 31;8(7):e69074. doi: 10.1371/journal.pone.0069074 (PMC3729808; doi:10.1371/journal.pone.0069074)
Supplement: Table S1 — Differences in the occurrence of the genera obtained by pyrosequencing between DNA and RNA samples. Positive values mean a higher prevalence at DNA level, negative values indicate a higher prevalence at RNA level. (DOCX) [file pone.0069074.s003.docx]

**Table 1S.** Differences in the occurrence of the genera obtained by pyrosequencing between DNA and RNA samples. Positive values mean a higher prevalence at DNA level, negative values indicate a higher prevalence at RNA level.

|  | **Not treated olives fermentation** | | | | | **NaOH treated olives fermentation** | | | | |
| --- | --- | --- | --- | --- | --- | --- | --- | --- | --- | --- |
|  | **Olives (days of fermentation)** | | | **Brines (days of fermentation)** | | **Olives (days of fermentation)** | | | **Brines (days of fermentation)** | |
| **Genus** | **3** | **8** | **90** | **8** | **90** | **3** | **8** | **90** | **8** | **90** |
| *Acinetobacter* |  |  |  |  |  |  |  |  | 6.04 |  |
| *Chromohalobacter* | 19.43 | -1.47 |  | -2.51 | -5.59 |  |  |  |  |  |
| *Citrobacter* |  |  |  |  |  | -6.63 | -33.64 | -17.25 | 1.32 |  |
| *Cobetia* |  |  |  |  |  |  |  |  | -4.39 |  |
| *Enterobacter* | 2.64 |  | 6.42 |  |  | -0.23 | 29.27 | 10.58 | 7.35 |  |
| *Escherichia* |  |  | 13.89 |  |  |  | 6.93 |  |  |  |
| *Flavobacterium* |  |  |  |  | -2.59 |  |  |  |  |  |
| *Halomonas* | 37.98 | -17.85 |  | -19.83 | -13.46 |  |  |  | 0.87 |  |
| *Klebsiella* |  |  |  |  |  | 1.88 |  | -2.23 | 0.47 |  |
| *Lactobacillus* |  |  | -15.44 |  | -8.83 |  |  | -4.28 |  | -16.26 |
| *Marinilactibacillus* |  |  |  | 23.57 | 30.73 |  |  | 7.03 |  | 9.74 |
| *Methylobacterium* | 7.88 | 10.64 |  |  |  |  |  |  |  |  |
| *Pantoea* | 17.95 | 2.32 |  |  |  | 0.03 |  |  | -13.91 |  |
| *Pseudomonas* | -54.22 |  |  |  |  |  |  |  |  |  |
| *Psychrobacter* | -5.85 |  |  |  |  |  |  |  |  |  |
| *Sphingomonas* | 7.91 |  |  |  |  |  |  |  |  |  |
| Others | -33.72 | 6.35 | -4.87 | -1.23 | -0.27 | 4.95 | -2.55 | 6.15 | 2.26 | 6.51 |
